# LCPan: efficient variation graph construction using locally consistent parsing

**DOI:** 10.1186/s13059-026-04088-w

**Published:** 2026-04-24

**Authors:** Akmuhammet Ashyralyyev, Zülal Bingöl, Begüm Filiz Öz, Kaiyuan Zhu, Salem Malikic, Uzi Vishkin, S. Cenk Sahinalp, Can Alkan

**Affiliations:** 1 Dept of Computer Engineering, Bilkent University, Ankara, 06800 Turkey; 2 Department of Computer Science and Engineering, University of California San Diego, La Jolla, 92093 CA USA; 3 Cancer Data Science Laboratory, Center for Cancer Research, National Cancer Institute, National Institutes of Health, Bethesda, 20892 MD USA; 4 University of Maryland Institute for Advanced Computer Studies (UMIACS), College Park, 20742 MD USA

**Keywords:** Locally consistent parsing, Genome representation, Variation graph

## Abstract

Efficient and consistent string processing is critical in the exponentially growing genomic data era. Locally Consistent Parsing (LCP) addresses this need by partitioning an input genome string into short, exactly matching substrings (“cores”), ensuring consistency across partitions. Compared to the popular sketching techniques, LCP produces fewer cores, enabling a more compact representation and faster analyses. Here, we present the first iterative implementation of LCP with Lcptools and introduce LCPan, an efficient variation graph constructor, which we show generates variation graphs >12$\times$ faster than vg, while using >13$\times$ less memory.

**Supplementary Information:**

The online version contains supplementary material available at 10.1186/s13059-026-04088-w.

## Background

Advances in genomic sequencing technologies and reductions in sequencing costs have significantly increased the production of sequencing data over the last two decades. These advancements have boosted the development of a plethora of computational methods for various use cases, including building genome assemblies [1], pangenome graph construction [2], phylogenetic analysis [3], and metagenome classification [4].

Due to the immense size of genomic sequence data, many of the computational approaches for various tasks such as genome assembly [1], metagenome distance estimation [5], read mapping [6], copy number variation (CNV) genotyping [7], and large-scale sequence database search [8] rely on representing, storing and indexing data as contiguous segments/substrings of length *k* (i.e., *k-mers*). However, indexing all possible k-mers consumes excessive memory. Alternatively, compressed (e.g., Burrows-Wheeler Transformation [9] and FM-index [10]) or hash-based set membership data structures (e.g., Bloom filters [11]) can be used to achieve smaller memory footprints at the cost of slower lookups [12].

To reduce memory requirements while retaining the advantages of constant-time querying, Roberts et al. proposed building a *sketch* of the data and indexing only a subset of k-mers, called *minimizers* [13], which can be broadly classified into two classes. The simpler class, called *universal minimizers*, includes only k-mers with a hash value less than a specified threshold [14], which are used, for example, in the rust-mdbg tool to quickly build minimizer-space de Bruijn graphs [15]. The second method finds the k-mers with the minimum hash values within all substrings of size *w* (i.e., *window*) over the input string. *Minimap2*, a state-of-the-art sequence alignment tool, uses this window-based approach to efficiently map long reads to the reference genomes [16]. An alternative approach to sampling substrings is Universal Hitting Set (UHS) [17], a set of k-mers included in every sequence of length *L*. The main drawback of this approach is that computing a minimal set for a given *k* and *L* is NP-hard [17], and thus approximation algorithms and heuristic methods are employed instead [17, 18].

To address accuracy loss due to sequencing errors, *strobemers* [19] were introduced to concatenate selected minimizers, thereby improving indexing and error tolerance. Alternatively, *syncmers* [20] select k-mers based on specific subsequence properties, offering better conservation, lower sampling density, and higher error tolerance compared to classical minimizers.

As an alternative to the aforementioned sketching techniques, Locally Consistent Parsing (i.e., LCP) [21, 22] proposes a string partitioning method that achieves consistent data reduction for building dictionaries, such as indexes. Sahinalp and Vishkin introduced LCP based on the Deterministic Coin Tossing (DCT) [23] technique, to efficiently construct suffix trees in parallel. LCP was also applied to pattern matching [21, 24] and edit distance computation [25]. A practical, non-iterative variant of LCP was implemented to boost the Lempel-Ziv algorithm for compressing biological sequences [26]. Later, locally consistent grammar-based text compression was proposed in [27]. Other applications of LCP, such as approximate string comparison and text indexing, can be found in [28–30]. However, except for the non-iterative variant for sequence compression [26], none of the previously proposed algorithms aim to process biological sequences, and only two open-source implementations are currently available [27, 30]. We note that these implementations are specifically for text indexing and compression, and they do not provide support for utilizing LCP for various use cases.

Locally Consistent Parsing (LCP) is a string processing technique that partitions and labels strings into nearly equal-length substrings, known as cores [21, 22]. We note that LCP is not a sketching method; however, the cores can potentially serve as an alternative to sketching. Unlike sketching techniques, LCP ensures that (1) the cores have a uniform positional distribution over the input string, (2) identical cores share the same labels, and (3) each character in the input string is included in at least one core. In this article, we introduce a generalized, iterative, and practical implementation of LCP, called Lcptools, providing an experimental analysis of the properties of the cores compared to minimizers, syncmers, MinHash sketches and UHS. Our analysis highlights that LCP cores are fewer in number and exhibit longer but more consistent distribution throughout the processed string. We also demonstrate the utility of LCP for variation graph construction using our novel algorithm LCPan (LCP-based Pangenome) that outperforms vg, the state-of-the-art variation graph constructor, in both running time ($>12\times$) and memory usage ($>13\times$), while demonstrating slightly better alignment accuracy using GraphAligner [31] on HiFi data. We note that both LCPan and vg generate variation graphs using a linear reference genome and a VCF file that contains genomic variation, where alternatives like pggb [32] and Minigraph-Cactus [33] build variation graphs using fully assembled contigs and scaffolds. We therefore limit our comparisons to only using vg for data consistency.

## Results

In this section, we provide our analysis of the properties of LCP cores at different levels, as well as LCPan efficiency. For our experiments, we used a server with an Intel Xeon CPU (2.2 GHz) with 56 cores (112 hyperthreads) and 768 GB of main memory.

### Properties of LCP cores

We analyzed the properties of the cores on the first fully assembled human genome (CHM13v2.0) generated by the Telomere-to-Telomere Consortium (T2T) [34]. We measured several metrics at each level up to level-8: 1) the number of identified cores, 2) the number of unique core labels, 3) the distances between the starting locations of cores, 4) the lengths of the underlying strings represented by the cores, and 5) the execution time to partition the input string up to the specified LCP level. Table 1 summarizes our findings and demonstrates that the entire genome can be represented by only 3.6 million cores at level 8, and each level nearly halves the number of cores.

As shown in Table 1, the total number of cores decreases progressively as the levels increase, from approximately 1.4 billion at level-1 to only 3.6 million at level-8. Between each successive level, the core count is reduced by a factor of 0.43. Because the reduction is consistent across successive levels, we can estimate the number of cores in each level for any given input string. This, in turn, enables us to calculate the memory footprint and the computational overhead for many genomic data applications. Conversely, the average distance between the starting coordinates of subsequent cores is 2.25 bp at level-1 and 854.87 bp at level-8. This change corresponds to a fold increase of approximately 2.25–2.34.25.34 between levels.

**Table 1:** The experimental results of LCP on CHM13v2.0

LCP level	1	2	3	4	5	6	7	8
Total # of Cores	1,385,807,259	595,674,177	253,384,737	108,531,321	46,426,965	19,885,308	8,514,640	3,646,315
Unique Cores	1,395	517,688	159,701,016	93,601,379	42,859,941	18,795,370	8,191,525	3,563,854
Exec. Time (sec)	42.22	25.75	12.10	5.04	2.10	0.87	0.36	0.14
Avg Distance	2.25	5.23	12.30	28.72	67.14	156.76	366.10	854.87
StdDev Distance	1.04	1.85	4.21	8.74	19.95	45.24	104.24	241.08
Avg Length	3.67	10.47	26.58	63.84	151.23	354.89	830.88	1,941.85
StdDev Length	1.01	1.90	4.32	8.83	19.49	43.02	97.57	222.96
Decrease in Core Count	0.44	0.43	0.43	0.43	0.43	0.43	0.43	0.43
Increase in Avg Length	3.67	2.85	2.54	2.40	2.37	2.35	2.34	2.34
Increase in Avg Distance	2.25	2.33	2.35	2.33	2.34	2.33	2.34	2.34
Total Size (GB)	30.98	13.31	5.66	2.43	1.04	0.44	0.19	0.08

We used Lcptools to generate LCP statistics using the CHM13v2.0 genome with a single iteration of DCT. Decrease in Core Cnt. is the proportional reduction in the number of cores compared to the previous level. Increase in Avg Len. is the proportional increase in the average core length compared to the previous level. Increase in Avg Dist is the proportional increase in the average distance between start positions of consecutive cores compared to the previous level. Notably, while the decrease in the core count is consistently 0.43, the increase in the average core length and the increase in the average distance converge to $1/0.43=2.33$

This observation empirically supports the theoretical analysis that $2 \le c \le 3$, as originally developed by Sahinalp and Vishkin [22]. We note that no k-mer-based sketching scheme (e.g., minimizers and syncmers) can guarantee fixed distances between consecutive substrings. Minimizers are only guaranteed to have a maximal gap of size *w*. We refer the reader to other work in the literature for an analysis of minimizer densities [35].

Next, we examined the average sequence length of cores at each level. At the first level, the average core length is 3.67, which aligns with expectations, since LMIN, LMAX, and SSEQ cores (with an alphabet of four letters) each span only three characters, whereas RINT cores are longer. The core lengths increase steadily at higher levels, exceeding 1.9 kb by level 8.

We then compared the LCP method against commonly used sketching methods, namely minimizers, syncmers, UHS, and MinHash, using the same input genome to assess the performance of each method regarding the number of selected k-mers and the distances between each selected k-mer. The LCP levels were selected as 2 and 3 because the average core length is similar to the commonly used k-mer sizes in practice. We selected $k=15$ and $w=10$ for the canonical minimizers as these are the default values in the popular minimizer-based alignment tool, Minimap2 [16]. For the syncmer analysis, we selected $k = 15$, $s=4$, and $t=0$, allowing s-mers to be located only at the beginning of the k-mer, based on the recommended settings in the original publication [20]. For the UHS analysis, we downloaded the pre-computed k-mer sets released by the authors for $k=13$ and L=20. Similarly, for MinHash, we analyzed the default Mash sketch sizes of 1,000 k-mers and 5 million k-mers ($k=31$), which roughly correspond to the number of cores in LCP level 8. We then mapped these k-mers to the CHM13v2.0 assembly and retained only those that matched exactly. Note that because of the repetitions in the genome, the number of observed k-mers in the genome is slightly higher than the k-mer counts in UHS and MinHash sketches.

As shown in Table 2, for $k=15$, we found over 665 million minimizers in CHM13v2.0, among which 92.6 million were unique. The syncmer count was smaller, at 371 million, with 58 million unique users. Since UHS is computed independently from a reference genome, the unique count was smaller, at 20.6 million. Still, when mapped to the genome, it returned 983.5 million locations, the highest value among all techniques tested. This is likely because the UHS set contained many k-mers with long homopolymers. MinHash is the coarsest-grained sketching method in our tests. As expected, it showed the fewest k-mers, a user-defined parameter. Similar to our LCP analysis, we calculated the average distances between each pair of minimizers, syncmers, UHS, and MinHash. On average, the distance between two consecutive minimizers was 4.69 nucleotides for $k=15$, whereas the distance between two consecutive syncmers was 8.40 nucleotides. UHS k-mers were more densely distributed in the genome, with an average distance of 3.16 nucleotides. Once again, MinHash demonstrated the widest range of distances between selected k-mers, with an average distance of 454.42 bp for the large sketch size (5 million) and 2.78 Mb for the default sketch size (1,000) in Mash. The cores in LCP level-2 were, on average, 10.47 bp, and in level-3, 26.58 bp; the former is slightly shorter, and the latter is slightly longer than the minimizer and syncmer lengths we considered. Although shorter, the number of level-2 cores was less than that of minimizers for $k=15$. Importantly, the number of unique level-2 cores is several orders of magnitude smaller than the minimizers or syncmers of any size, thanks to consistent identification of cores using LCP. The number of level-3 cores is less than all the alternatives we considered, except MinHash. Perhaps most importantly, among all the other sketches, LCP deviates proportionally less from the average distance. Higher LCP levels show even lower numbers of cores, therefore reducing the computational cost for storage, indexing, and query (Table 1).

**Table 2:** Properties of minimizers, syncmers, and UHS compared to LCP in CHM13v2.0

Metric	Minimizer	Syncmer	UHS	LCP-2	LCP-3	MinHash-1K	MinHash-5M
K-mer size†	15	15	13	10.47	26.58	31	31
Window size‡	10	−	20	-	-	-	-
S-mer size	$-$	4	-	-	-	-	-
Total k-mers	665.1M	371.1M	983.5M	595.7M	253.4M	1.1K	6.9M
Unique k-mers	92.6M	58M	20.6M	0.5M	159.7M	1K	5M
Avg Distance	4.69	8.40	3.16	5.23	12.30	2.78M	454.42
StdDev Distance	3.15	8.20	1.36	1.85	4.21	3.2M	463.73

†We show the average core length for LCP, and ‡ *L* for UHS. We also provide the statistics for MinHash using sketch sizes of 1,000 and 5,000,000

We note that finding minimizers and syncmers requires only a linear scan of the input string and simple lexicographic comparison, and therefore is faster in practice than identifying cores in LCP. Computing a minimum-size UHS, on the other hand, is NP-hard [17]; thus, the UHS k-mer sets can only be approximated. However, the granularity of sketches generated by LCP can be adjusted across different levels. Therefore, depending on the specific use case, longer but fewer or shorter but more frequent cores can be used. On the other hand, higher LCP levels are constructed by parsing lower levels; therefore, cores across several levels can be computed in a single pass over the input string. Similar flexibility from minimizers and syncmers can be achieved only by changing the parameters *k*, *w*, and *s*, which will require multiple passes over the input string and incur significant bottlenecks in both the sketching method’s runtime and memory footprint. However, we leave a rigorous comparison of LCP with sketching methods for read mapping as future work.

### Properties of LCPan graphs and construction performance

We constructed variation graphs, with varying LCP levels, using all chromosomes of the human reference genome (GRCh38) as the backbone and the genomic variations released by the Human Pangenome Reference Consortium [36] (Table 3). We first evaluated LCPan’s performance at different LCP levels. We observed a consistent decrease in the number of segments in LCPan, from 408 million at level-4 to 67 million at level-7. The number of links followed a similar pattern, decreasing from 423 million to 82 million between levels 4 and 7. The average segment length increased from 26.19 bp to 158.32 bp as the LCP level increased, while the standard deviation of the segment lengths rose from 11.81 bp to 184.45 bp. Reducing the number of segments and links reduced memory usage from 8.34 GB to 5.61 GB. We also observed a similar decrease in the final output size in the GFA and rGFA formats up to level-7. vg generated a variation graph with an average segment length of 28.23 bp, comparable to LCP level-4, and a standard deviation of 9.67 bp. However, it used significantly more memory at 114.56 GB. LCPan was >12.35$\times$ faster than vg at level-4 while using 13.74$\times$ less memory, with additional improvements at higher LCP levels. Note that vg cannot process large inputs, particularly for chromosomes that contain tens of millions of base pairs^1^ and with VCF files that contain millions of variations. To manage such large data sets, we followed the vg user manual to process the genome into segments, then merged the outputs using the *vg combine* command. The construction of variation subgraphs took 35 minutes and used 9.18 GB of memory, while merging required an additional 47.5 minutes and 114.56 GB of memory. In addition to the construction time, vg requires an additional $\sim$11 minutes and 91 GB of memory to convert the output graph to GFA format.

**Table 3:** Experimental evaluation of LCPan and VG using the HPRC data set on GRCh38

Metric	LCPan(4)	LCPan(5)	LCPan(6)	LCPan(7)	VG
Total # Segments	408,484,289	196,920,897	106,363,844	67,566,416	490,391,209
Total # Links	423,677,755	212,114,363	121,557,310	82,759,882	519,013,255
Avg. Seg. Len. (bp)	26.19	54.32	100.57	158.32	28.23
StdDev Seg. Len. (bp)	11.81	32.00	80.57	184.45	9.67
Exec. Time (sec)	401.61	320.67	285.79	299.27	4958.44
Peak memory (GB)	8.34	6.67	5.95	5.61	114.56
Output r/GFA (GB)	44.24/28.11	26.61/18.94	19.10/15.05	15.89/13.40	-/33.83

We compared graph construction metrics between LCPan and VG using the HPRC data set [36] on GRCh38. The table shows the total number of segments, total number of links, execution time, average segment length, standard deviation of segment length, memory usage (RSS), and output size for different LCP levels of LCPan and VG. Graphs are constructed using all chromosomes

**Table 4:** Yeast pangenome construction using 100 strains with LCPan, VG, and VariantStore

Metric	LCPan(5)	VG	VariantStore$^*$
Total # Segments	252,142	437,196	73,080
Total # Links	276,486	461,631	97,440
Avg. Seg. Len. (bp)	47.97	27.67	165.51
StdDev Seg. Len. (bp)	31.86	10.34	10.87
Exec. Time (sec)	0.52	2.25	9.61
Peak memory (MB)	34.63	84.17	100.80
Output r/GFA (MB)	14	20	N/A

Comparison of graph construction metrics between LCPan (level 5), VG, and VariantStore using 100 yeast strains [37] on *S. cerevisiae* assembly R64 (GCA_000146045). $^*$ VariantStore values represent the overall values for all chromosomes

As an alternative to the human genome, we repeated the graph construction analysis using 100 yeast strains [37] (Table 4). We built variation graphs with LCPan, vg, and VariantStore [38]. We note that VariantStore does not generate a mappable variation graph; rather, it primarily indexes genomic variants across multiple genomes. Our observations were in line with the human genome experiment, albeit to a lesser degree, where LCPan showed a 4.33$\times$ speedup over vg and an 18.48$\times$ speedup over VariantStore, with improvements of 2.43$\times$ and 2.91$\times$ in peak memory usage, respectively.

### Scaling to multiple threads

**Fig. 1:**
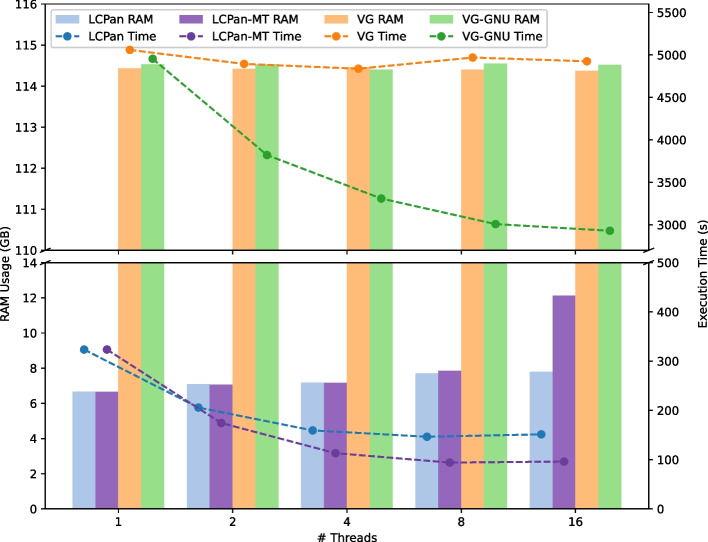
Multi-thread scaling analysis of LCPan and vg using HPRC data on GRCh38. Memory and run time of graph construction using different numbers of threads. LCPan and vg were run using their native multithreading implementations. vg was additionally parallelized using GNU Parallel by distributing single-threaded processes on chunks across concurrent jobs (VG-GNU)

LCPan first processes the reference genome to build the backbone graph sequentially and then assigns the set of variations to multiple threads to amend the graph. The partition consistency provided by LCP enables LCPan to efficiently scale computation into multiple threads (see Variation graph construction with LCPan section for details). In the default threading scheme, parallelism is applied during the execution of DCT and variation integration into the graph, while LCP-core identification remains largely sequential. When the –full-parallel option is employed, LCPan additionally executes LCP-core identification by chromosome-level parallelization. This execution mode is referred to as LCPan-MT in the Fig. 1.

To evaluate the parallelization performance, we constructed the same variation graph using different numbers of threads for both LCPan and vg (Fig. 1). We note that vg documentation recommends parallel construction in two ways: 1) a command-line parameter (vg construct -t), and 2) by partitioning the genome and VCF file into chunks and distributing the task across multiple threads using the GNU Parallel tool [39]. We have used both methods for vg in our evaluation.

We observed that the memory usage was not significantly affected across different thread settings for both LCPan and vg. In contrast, the chromosome-level parallelization in LCPan-MT increases peak memory from $\sim$8 GB to $\sim$12 GB at higher thread counts because more chromosomes are processed concurrently. This design deliberately trades a modest increase in memory usage for improved runtime performance at higher levels of parallelism. Meanwhile, LCPan speed improved up to 8 threads, and adding more threads did not improve run time.

For vg, we observed that the native command-line parameter had no effect on improving efficiency, whereas the GNU Parallel-based technique provided speedups of up to 16 threads. In comparison, LCPan exhibits better scalability in both cases.

### Alignment accuracy

We next evaluated the accuracy of read mapping to variation graphs generated by LCPan and vg, using various read (PacBio HiFi and ONT) and variation graph data sets. As outlined in the introduction, a direct comparison with pggb and Minigraph-Cactus is methodologically inconsistent because LCPan and vg construct variation graphs by augmenting a linear reference with variants from a VCF file, whereas pggb and Minigraph-Cactus build graphs *de novo* using multiple whole-genome assemblies. We therefore limited our comparisons to only vg. We performed the experiments with chromosomes 1, 10, and 22 (GRCh38), which provide a range of lengths and repeat/duplication complexity.

We first constructed both LCPan and vg variation graphs using only chromosomes 1, 10, and 22 (GRCh38) and the variants released by the Human Pangenome Reference Consortium (HPRC) [36]. vg constructed the graph in 6 m 18 s using 12.91 GB of memory, while LCPan constructed the graph in only 2 m 16 s using 2.17 GB of memory. We set the k-mer size in the vg graph to 64 and the LCP level to 5 for LCPan to make the average segment sizes (i.e., lengths of the sequences represented in nodes) comparable. The resulting graphs included segments of average lengths 56.25 bp and 55.17 bp for LCPan and vg, respectively.

Next, we aligned both PacBio HiFi and ONT reads generated from the HG002 sample by the Telomere-to-Telomere (T2T) Consortium (v1.0 assembly polishing/mapping data) [40], to the constructed variation graphs. Since LCPan is focused solely on variation graph construction, and vg is optimized for short read data [41], we used GraphAligner [31] with 96 threads in our mapping experiments for both types of graphs.

**Table 5:** GraphAligner alignment results for PacBio HiFi and ONT reads aligned on LCPan and VG graphs

Graph	LCPan			VG		
Dataset	PacBio HiFi$^\ddag$	PacBio HiFi$^\dag$	ONT$^\ddag$	PacBio HiFi$^\ddag$	PacBio HiFi$^\dag$	ONT$^\ddag$
Input Reads	820,665	5,764,123	281,312	820,665	5,764,123	281,312
Reads w/align.	820,665	5,764,122	281,306	820,665	5,764,123	281,308
Align. Time (hh:mm:ss)	06:49:06	63:46:26	06:19:06	08:12:07	91:34:26	07:42:47
Memory (GB)	82.01	220.08	114.57	83.08	411.91	103.29
Precision	0.40	0.49	0.41	0.38	0.38	0.41
Recall	0.70	0.53	0.70	0.72	0.72	0.71
F1	0.51	0.51	0.52	0.50	0.50	0.52

The average lengths of PacBio HiFi and ONT reads are 15,955 bp and 47,000 bp, respectively, corresponding to $\sim$30$\times$ depth of coverage. Precision, recall, and F1 values are comparable across all tests, while LCPan-based alignment using GraphAligner is $\sim$1.2$\times$ faster. Alignments to $^\ddag$ chromosomes 1, 10, and 22 only, and to $^\dag$ the full genome. Small-scale tests (chromosomes 1, 10, 22) used only those that are known to map to their respective chromosomes, while the full-genome tests used the entire read data sets

We used the structural variation (SV) call sets released by the the Genome in a Bottle Project [42]^2^ using the pbsv tool [43], as a proxy to calculate alignment accuracies. Our evaluation involved calculating precision, recall, and F1-scores, with a tolerance of a ±100 bp margin around the SV breakpoints. To ensure a fair comparison, we also generated SV calls using the pbsv tool, and restricted the variations to match the ground truth. Table 5 summarizes the mapping results. Nearly all reads were aligned to the vg and LCPan graphs across two data sets. However, we observe that GraphAligner completed alignments on the LCPan graph $\sim$1.2$\times$ faster than on the vg graph, though it required slightly more memory on the ONT dataset. Overall, performance metric values for accuracy across different data sets using LCPan and vg graphs remained consistent.

Finally, we repeated our read mapping test using the full variation graphs we created across all chromosomes in GRCh38. Since GraphAligner requires substantial time for read mapping, we limited our test to only the PacBio HiFi data sets. In this test, we observed that GraphAligner was 1.4$\times$ faster using the LCPan graph compared to the vg graph, with 1.87$\times$ less peak memory (Table 5).

## Discussion

In this paper, we showed that LCP provides a consistent, uniform, and efficient approach for genomic string partitioning. We also showed that LCP improves computational efficiency and parallelizes the construction of the variation graph. One of the key features of the LCP technique is its flexibility in selecting levels, allowing the adjustment of core granularity based on the requirements of specific use cases. For example, middle-level (i.e., 4–5) can be more appropriate for genomic data compression, where large CNV discovery could be achieved using higher levels. LCP cores can also be used in place of k-mers or minimizers for genome assembly with long, accurate reads. The relationships between levels and the length and distance properties at each level could also be utilized; for example, a hierarchical strategy can be used to align long sequences. However, analyzing “best practices” for LCP level selection for each potential use case requires rigorous experimentation, which we leave for future work.

We also demonstrated that using LCP, our variation graph construction method, LCPan, significantly reduces memory consumption while maintaining high computational efficiency. Partition consistency provided by LCP enables better parallelization and more efficient resource utilization, leading to lower memory usage and faster execution times. Experimental results show that LCPan consistently outperforms vg in execution speed and scales well with an increasing number of threads.

While LCPan currently only implements the graph-building step, vg is an all-inclusive tool that builds the graph, aligns reads, and genotypes variants. Although vg supports multi-threading, its performance did not scale efficiently with increasing thread count. On the other hand, even without indexing and cache optimizations, LCPan surpasses vg in graph construction efficiency while reducing memory usage and execution time. This demonstrates the inherent efficiency of LCP-based construction of variation graphs. However, a formal analysis for alignment accuracy remains as future work.

## Conclusions

Although originally developed more than 30 years ago, Locally Consistent Parsing has remained limited in practical use. LCP applies, in theory, to various problems arising from sequence analysis, such as edit distance approximation [28], string embeddings [25], memory-efficient text indexing [29], and compression [27]. Here, we provide an API that enables researchers to fully exploit the LCP method across different use cases. Our implementation can easily be extended for non-DNA alphabets, enabling the processing of, for example, protein sequences. Further research using LCP, made practical through Lcptools, will potentially prove the efficiency and accuracy of various algorithms and impact biomedical research.

## Methods

The key properties of LCP have been formally defined earlier in [22]:

### Definition 1

*Partition Consistency: Given an input string S, suppose that a substring* $X_i$ *starts at position* *i* *and* $X_j$ *starts at position* *j*. *If* $X_i=X_j$, *then with a possible exception of left and right margins*, $X_i$ *and* $X_j$ *are partitioned in the same way*.

### Definition 2

*Labeling Consistency: All cores that consist of the same characters are assigned the same labels*.

Note that these properties of LCP supersede the *window guarantee* provided by sketching techniques since all of the input string is represented within cores.

Below, we describe a simple variant of LCP, expressed in terms of four rules, that identifies cores in an input string over a given alphabet (e.g., the 4-letter DNA alphabet). We will later show how to apply LCP iteratively using DCT in Iterative application of locally consistent parsing section.

1. *Local Minimum Rule (LMIN):* A substring $w, |w|=3$, $w=xyz$, is a core, if the middle character *y* is a *proper* local minimum (w.r.t. the lexicographic ordering of the characters). Specifically, for $x \ne y$ and $y \ne z$, the middle character *y* must satisfy $x> y$ and $y < z$.
2. *Local Maximum Rule (LMAX):* A substring $w, |w|=3$, $w=xyz$, is a core, if the middle character *y* is a proper local maximum, and neither *x* nor *z* is a local minimum. Specifically, for $w=xyz$ that appears within the superstring *sxyzt*, the middle character *y* satisfies $x < y$, $y> z$, and additionally $s \le x$ and $z \ge t$.
3. *Repetitive Interior Rule (RINT):* A substring $w, |w|>3$ is a core if all its characters except the first and the last characters are identical. Specifically, for $w=xy^iz$, where $i> 1$, $x\ne y$, and $y\ne z$, the substring *w* satisfies the condition of being a core.
4. *Stranded Sequence Rule (SSEQ):* A substring $w, |w|>2$ is a core if its characters are either strictly increasing or decreasing w.r.t. the lexicographic order, and only the first and last characters are part of either a *LMIN*, *LMAX*, or a *RINT* cores. Specifically, if $w=xyz {a_1 \dots a_n} klm$, where $n\ge 1$ and *xyz* and *klm* are identified as cores and $z<a_1<\dots<a_n<k$ or $z>a_1>\dots>a_n>k$, then $z {a_1 \dots a_n} k$ is a core (SSEQ type).

**Fig. 2:**
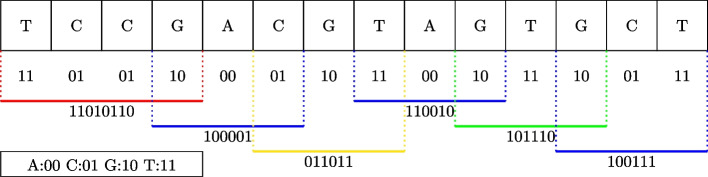
Processing a string using LCP. Here, blue underlines the core that satisfies the Local Minimum core, green represents a Local Maximum core, red corresponds to a Repetitive Interior core, and yellow denotes a Stranded Sequence core

Figure 2 depicts an example of the cores identified in a short DNA sequence. As the cores are identified, the two-bit encoding of the underlying characters (i.e., A = 00, C = 01, G = 10, T = 11) is used to form the core’s *bitstream*. Note that we use the term *core alphabet* to refer to the bitstreams that form the cores, specifically the DNA encoding used in the first LCP iteration.

Given any input string^3^, LCP ensures that the distances between consecutive cores are small. We now show that the cores identified through the rules above satisfy the *Contiguity Property* and the *Adjacency Property*. We provide the lemma definitions below and the formal proofs in Additional file 1.

### Lemma 1

Contiguity Property: There are no gaps between any pair of consecutive cores identified by LCP.

The contiguity property guarantees that the cores fully represent the input string and all characters in the input are included in at least one core.^4^

### Lemma 2

Adjacency Property: within a substring of length 3, at most 2 characters can be the starting positions of the cores.

The adjacency property dictates that for $w=xyzlmn$, if *xyz* is a core, the next two cores cannot start simultaneously at *y* and *z*. As the number of cores that start in a window of size 3 cannot exceed 2, we can assert that, in the worst case, the total number of cores will be bounded by 2*n*/3, where *n* is the length of the input string. The worst case occurs, for example, in $S=gcggcggcg\dots gcg$, where $c < g$. In this sequence, the number of LMIN cores (e.g., *gcg*) will be *n*/3, and the number of RINT cores (e.g., *cggc*) will be $n/3-1$, resulting in a total of $2n/3-1$ cores. However, if LMAX and SSEQ cores are also identified in the string, they are positioned farther apart, resulting in fewer cores.

### Iterative application of locally consistent parsing

Our goal is to apply LCP **iteratively**, so that at higher levels, cores become longer, the distance between consecutive core start positions increases, and the total number of cores decreases, all by approximately a constant factor per level. A direct attempt to do this by reapplying the LCP rules to the raw **bitstream representations** of level-*i* cores is problematic: once we move beyond the DNA alphabet, the “symbols” (core bitstreams) effectively come from a much larger, variable-length alphabet, and applying the LCP core-identification rules on such an alphabet can lead to **uncontrolled growth** in spacing and loss of the even spacing properties ensured by the LCP lemmas at the base level.

**Fig. 3:**
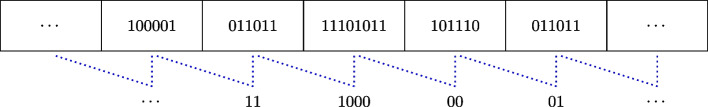
DCT for reducing bitstreams to a new alphabet. Each block above corresponds to the bitstream of a core. The DCT compares each core’s bitstream to its left neighbor to form a shorter alphabet. E.g., the least significant bit of the core bitstream 11101011, which differs from its left neighbor 011011 at the fourth index (counting from the right and starting at index 0). The value of this bit is 0. Therefore, DCT replaces the core bitstream 11101011 with the concatenation of the bits of 4, and the value of the differing bit, resulting in 1000. The figure shows the reduced alphabet inferred by the DCT for each core bitstream, on which LCP can be applied

To keep iterative parsing well-behaved, we incorporate **Deterministic Coin Tossing (DCT)** when constructing levels $i>1$. DCT maps each level-*i* core bitstream to a shorter code over a reduced alphabet by comparing it to its **immediate left neighbor** (Fig. 3). Concretely, let $b_{j-1}$ and $b_j$ be the (right-aligned) bitstreams of two consecutive level-*i* cores. DCT finds the **least significant position** *t* (counting from the right, starting at 0) where $b_{j-1}$ and $b_j$ differ, and replaces $b_j$ by a new code that concatenates (i) the binary representation of *t* and (ii) the value of $b_j$ at position *t*. If the original bitstreams have length *k*, then $t\in {0,\ldots ,k-1}$ and the DCT code has length $\lceil \log k\rceil +1$ bits (the index plus one bit for the value). This reduction controls the effective alphabet size of the sequence on which LCP is applied, so that the resulting “string of reduced symbols” continues to satisfy the contiguity/adjacency-style constraints needed for iterative LCP.

**Fig. 4:**
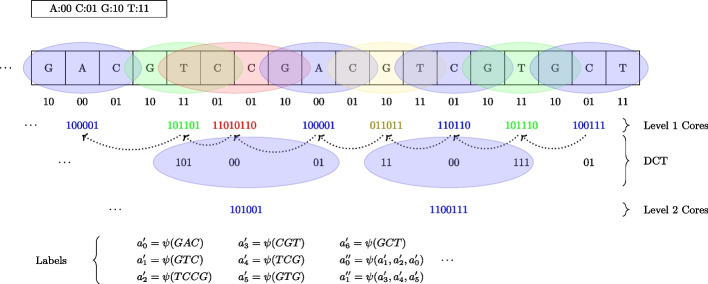
Overview of the LCP with DCT and labeling paradigm

After DCT, we apply the standard LCP core rules to the resulting reduced-symbol sequence to obtain **level-**(i+1) cores. If a level-($i+1$) core *x* is the concatenation of consecutive level-*i* cores $y,z,w,\ldots$ (i.e., $x=yzw\ldots$), then the level-($i+1$) bitstream for *x* is defined as the concatenation of the corresponding DCT codes $y',z',w',\ldots$. Because DCT is deterministic and depends only on adjacent core bitstreams, repeated occurrences of the same block of level-*i* cores yield the same reduced representation **up to boundary effects**, which is sufficient to support locally consistent parsing at higher levels.

Equipped with DCT, each LCP level reduces the number of cores while increasing both the **average core length** and the **average distance between consecutive cores** by a constant factor *c*. From the adjacency bound at each level, *c* is at least 3/2 in the worst case; empirically, on the human genome, we observe $c\approx 2.34$ (Table 1). Thus, at level-*i*, we expect on the order of $n/c^i$ cores representing substrings of average length $\Theta (c^i)$, and the average distance between consecutive core start positions is $\Theta (c^i)$. Each level can be computed in time linear in the length of the level’s representation, and because the representation shrinks geometrically across levels, the total time to compute all levels is linear in the original input length.

Our implementation applies **only one round of DCT** as a pragmatic tradeoff between LCP’s guarantees and the efficiency of the “anchor”. Each additional DCT round requires increasing the **core-substring length (and thus overlap between consecutive cores)** to preserve the key property that **core-ness of a substring is context independent** (a core remains a core wherever it occurs). This added overlap between consecutive cores increases redundancy and reduces sparsity. As we have demonstrated, a single round of DCT already yields a stable, well-behaved empirical distribution of distances between consecutive cores on the human genome, so further rounds offer limited benefit relative to their cost. In rare cases, a single round can create a short stranded region not covered by any core substring; however, empirically, such regions are highly infrequent and do not measurably affect the overall distribution of distances between consecutive cores.

### Labeling paradigm of LCP cores

A key concern in the LCP technique is the process of assigning labels to LCP cores. This step is essential, as the assigned labels represent the underlying string and must be as distinct as possible. To achieve this, we follow the following approach to labeling cores. For a core *x* at level $i>1$, and if it is composed of level-$(i-1)$ cores *y*, *z*, $w,\ldots$, then the label for *x* is assigned as $x''= \psi (y'', z'', w'', \ldots )$. Here, $\psi$ is a hash function designed to combine the labels $y''$, $z''$, $w'', \ldots$ to ensure the distinctiveness of the new core label $x''$. For labeling level-1 cores, the labels are directly derived from the characters of the underlying string, ensuring that each character or substring is uniquely represented.

Figure 4 illustrates these high-level concepts of LCP, DCT, and labeling, to provide a comprehensive visual summary of the overall process.

**Fig. 5:**
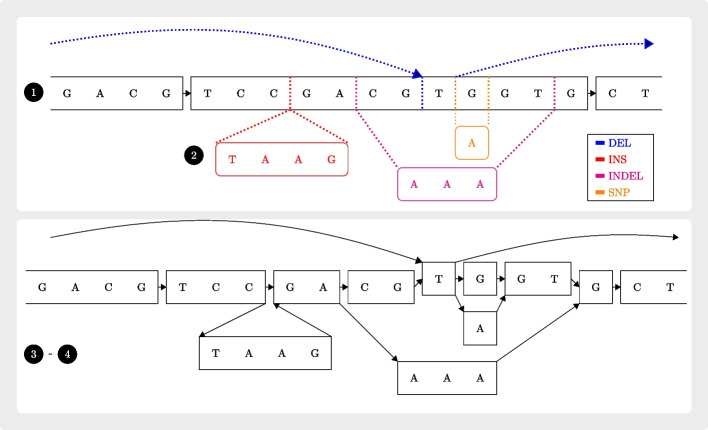
Overview of the LCPan method

### String processing with Lcptools

We have developed Lcptools as a C-based application programming interface (API) that implements the LCP method, which iteratively processes input strings and generates cores at multiple levels. Lcptools is primarily designed for processing genomic sequences as it assumes the input strings are generated from the DNA alphabet (i.e., $\Sigma =\{A,C,G,T\}$). Lcptools also includes functions for saving and loading the cores for later use. To enhance the flexibility and usability of Lcptools, we provide several options for performing LCP, including custom alphabet encoding. Finally, labels are assigned to cores using MurmurHash [44] as $\psi$, ensuring efficient and consistent hashing for labeling (Fig. 4).

### Variation graph construction with LCPan

To highlight the efficiency and accuracy gained using the LCP technique for string processing, we developed LCPan, a variation graph construction tool that leverages the Lcptools API to partition strings rapidly. The state-of-the-art variation graph construction tool, vg, internally constructs the graph, performs all computations within it, such as simplification, and then serializes the result, which ultimately leads to high resource consumption. Unlike vg, LCPan improves memory utilization, resulting in lower storage and memory requirements while maintaining near-uniform partitioning.

Figure 5 illustrates LCPan’s steps for graph construction. Using the selected LCP level, we partition the initial linear genome into LCP cores per chromosome (step ❶). The initial linear genome yields a simple linear “backbone” graph in which nodes represent cores, and edges connect pairs of consecutive cores at the associated LCP level. For each variation in the input VCF file, we apply the respective operation for the variation (step ❷) to each of the associated sets of LCP cores in this graph. Each branch created between the consecutive LCP cores generates an alternative path in the graph. If the offset of a variation overflows the current LCP core’s range, we reassign it to the next appropriate LCP core. LCPan then further partitions the core (step ❸) to integrate additional variations from alternative haplotypes into the graph (step ❹), which, in its final form, is referred to as the variation graph. Finally, at both ends of the sequences, we allocate separate segments to fully represent the reference sequence. Note that there might be at most two such segments in a given ungapped sequence.

The fundamental strength of LCPan lies in the guarantees provided by Locally Consistent Parsing (LCP), particularly regarding partition consistency. This consistency is essential, as it ensures that the partitioned substrings maintain near-uniform lengths. By sequentially processing variation files stored in sorted order, variations within the offsets of LCP cores can be organized and processed concurrently and independently in batches. Moreover, consistent partitioning and labeling facilitate rapid matching, as LCP ensures that sequences are parsed identically and independently, even when separated by megabases. Furthermore, higher LCP levels lead to longer cores and fewer partitions during parsing. Each partition then contains more variations that must be processed together, resulting in more concentrated work per execution unit. Consequently, performance initially improves with the addition of more threads, but after a certain threshold (e.g., 8 threads), the benefit of adding more threads diminishes due to the limits of parallelization.

## Supplementary Information

Additional file 1. Proofs for the contiguity and adjacency properties.

## Data Availability

An open source (BSD 3-Clause License) implementation of Lcptools is available at https://github.com/BilkentCompGen/lcptools, and LCPan is available at https://github.com/BilkentCompGen/lcpan. The versions used in this manuscript are also archived at Zenodo: https://doi.org/10.5281/zenodo.19528293 (Lcptools) and https://doi.org/10.5281/zenodo.19528340 (LCPan). All data used in this study are publicly available. Human pangenome variation data from the Human Pangenome Reference Consortium (HPRC) (https://s3-us-west-2.amazonaws.com/human-pangenomics/pangenomes/freeze/freeze1/pggb/vcfs/) [45] were used for large-scale variation graph construction and comparison of vg and LCPan. The Saccharomyces cerevisiae S288C reference genome is available at the European Nucleotide Archive (accession GCA 000146045.2) [46] and yeast variation dataset (https://github.com/daskelly/yeast100genomes) [47] were used for additional graph construction experiments and benchmarking. Sequencing datasets, including PacBio HiFi (https://s3-us-west-2.amazonaws.com/human-pangenomics/T2T/HG002/assemblies/polishing/HG002/v1.0/mapping/hifi_revio_pbmay24/) [48] and ONT ultra-long reads (https://s3-us-west-2.amazonaws.com/human-pangenomics/T2T/HG002/assemblies/polishing/HG002/v1.0/mapping/ont_r10_ul_dorado/) [49], were used for sequence-to-graph alignment experiments. Structural variant calls from the Genome in a Bottle Consortium (https://ftp-trace.ncbi.nlm. nih.gov/ReferenceSamples/giab/data/AshkenazimTrio/analysis/PacBio_pbsv_05212019/) [50] were used for benchmarking.

## Footnotes

1 https://github.com/vgteam/vg/issues/4152

2 https://ftp-trace.ncbi.nlm.nih.gov/ReferenceSamples/giab/data/AshkenazimTrio/analysis/

3 From this point on, we will use the terms *string* and *sequence* interchangeably.

4 This may be violated by a small number of characters at the extreme ends of the input string.
