# Supplementary material for: LCPan: efficient variation graph construction using locally consistent parsing
Source: Genome Biol. 2026 Apr 24;27:197. doi: 10.1186/s13059-026-04088-w (PMC13270696; doi:10.1186/s13059-026-04088-w)
Supplement: Supplementary file 1 — Additional file 1. Proofs for the contiguity and adjacency properties. [file 13059_2026_4088_MOESM1_ESM.pdf]

Additional File 1: Proofs

Contiguity and Adjacency Properties

Lemma 1. *Contiguity Property: no gaps exist between any pair of consecutive cores identified by LCP.*

The contiguity property guarantees that cores fully represent the input string and prevents any characters in the input string from not being included in any core. We prove that LCP satisfies this property below.

Proof. Given a substring $w = xyzlmn$, one of the following must be correct.

1. If xyz is an LMIN core, then the substring zlm or lmn may satisfy the LMAX rule ($y < z < l$ and $l > m \geq n$; or $y < z < l < m$ and $m > n$), assuming n is not the middle character of another LMIN core. On the other hand, if the lexicographic order of subsequent characters increases (i.e., $y < z < l < m < n$), the SSEQ rule will apply after LMIN until the downstream of the input string includes an LMIN, LMAX, or RINT core. Finally, if $z = l$ or $l = m$, a RINT core will follow an LMIN core.
2. If xyz is an LMAX core, then the substring zlm or lmn may satisfy the LMIN rule ($y > z > l$ and $l < m$; or $y > z > l > m$ and $m < n$). Alternatively, if the lexicographic order of subsequent characters decreases (i.e., $y > z > l > m > n$), the SSEQ rule will apply after LMAX until the downstream of the input string includes an LMIN, LMAX, or RINT core. Finally, if $z = l$ or $l = m$; a RINT core will follow an LMAX core.
3. If $x\overline{y}z$ is a RINT core (i.e., $x\overline{y}z = xy^iz, i > 1$), then the substring zlm or lmn may satisfy the LMIN rule ($y > z > l$ and $l < m$; $y > z > l > m$ and $m < n$). The RINT substring $x\overline{y}z$ can also be followed by an LMAX core where zlm or lmn may satisfy the LMAX rule ($y < z < l$ and $l > m$; or $y < z < l < m$ and $m > n$). If the lexicographic order of subsequent characters decreases (i.e., $y > z > l > m > n$), or increases (i.e., $y < z < l < m < n$), an SSEQ core will follow the RINT core until one of the LMIN, LMAX, or RINT rules is satisfied. If $z = l$ or $l = m$, the RINT core will be followed by another RINT core.
4. By definition, an SSEQ core shares characters with its neighbor, concluding the correctness of the Contiguity Property.

□

We showed in Lemma 1 that a string can be fully represented without gaps using cores. Below, we show that there is an upper bound on the number of potential cores for a given string; this has implications for the efficiency of string processing and indexing. In fact, we demonstrate that the number of cores is a constant factor smaller than the length of the input string after LCP processing[9]. For that, we define the *Adjacency Property* as follows.

Lemma 2. *Adjacency Property: within a substring of length 3, there could be at most 2 characters that could be the starting positions of cores.*

Similar to Lemma 1, the correctness of Lemma 2 can be proven by analyzing the possible placement of cores in substrings of length three.

[9] The number of cores is further reduced after each iterative call to LCP; the cores identified in iteration i are called level i cores.

Proof. Given a string $w = xyzlmn$, one of the following must be correct.

1. If xyz is an LMIN core, then zlm or lmn may be an LMAX core (i.e., $z \neq l$ and $l \neq m$). By definition, no local minimum can exist adjacent to an LMAX core. Hence, they do not overlap for more than one character (i.e., an LMAX core cannot start with y). On the other hand, the closest RINT core after an LMIN core in w can be in $yzlm$, if $z = l$. The next core after $yzlm$ can only start with l since the prefix of no core may be a repeat. Similarly, by definition, an SSEQ core may start only at or after z.
2. If xyz is an LMAX core, the closest LMIN core may only start at z. The closest RINT core could start at y if $z = l$, but in that case, no other core may start at z for the same reason outlined above. Again, the same reasoning applies to the next SSEQ core that follows an LMAX core.
3. If xy^iz is a RINT core ($i > 1$), the closest possible cores may start after i characters. For example, if $w = xyyznm$, then $xyyz$ is a RINT core, and the next LMIN, LMAX, or SSEQ core may start at $yzn \ldots$.
4. If xyz is an SSEQ core, the closest possible core may start after the third character (i.e., it may be zlm).
